# Mindfulness and autonomous sensory meridian response (ASMR)

**DOI:** 10.7717/peerj.5414

**Published:** 2018-08-07

**Authors:** Beverley K. Fredborg, James M. Clark, Stephen D. Smith

**Affiliations:** 1 Department of Psychology, Ryerson University, Toronto, ON, Canada; 2 Department of Psychology, University of Winnipeg, Winnipeg, MB, Canada

**Keywords:** ASMR, Autonomous sensory meridian response, Mindfulness, Toronto mindfulness scale, Mindful attention and awareness scale

## Abstract

**Background:**

Autonomous sensory meridian response (ASMR) is a perceptual phenomenon in which specific audiovisual stimuli frequently elicit tingling sensations on the scalp and neck. These stimuli (“ASMR triggers”) are typically social in nature (e.g., watching someone brush their hair, hearing whispering,) and often elicit a calm and positive emotional state that may last up to several minutes. ASMR experiences phenomenologically overlap with mindfulness; however, no research has directly examined how mindfulness might relate to ASMR.

**Methods:**

In the current study, 284 individuals with ASMR completed the Toronto Mindfulness Scale (TMS), the Mindful Attention and Awareness Scale (MAAS), and a questionnaire examining ASMR experiences. Age- and sex-matched control participants were asked to view two ASMR-eliciting videos to ensure that they did not experience tingling sensations associated with ASMR; they then completed the TMS and MAAS questionnaires.

**Results:**

When compared with matched controls, individuals with ASMR generated significantly higher scores on the MAAS, a global measure of mindfulness, as well as significantly higher scores on the Curiosity subscale of the TMS.

**Conclusions:**

These results suggest that the sensory-emotional experiences associated with ASMR may be partially explained by a distinct subset of characteristics associated with mindfulness.

## Introduction

Autonomous sensory meridian response (ASMR) is a perceptual phenomenon in which specific stimuli, known as “ASMR triggers,” frequently elicit tingling sensations on the scalp, neck, and shoulders, often spreading to the body’s periphery. These sensations are generally accompanied by a sense of calm as well as by positive affect (Barratt & Davis, 2015). The triggers that elicit these experiences vary widely between individuals and may be auditory, visual, tactile, and/or olfactory in nature. Despite their heterogeneity, the most popular ASMR triggers tend to be audio–visual; a recent survey study of individuals with ASMR found that whispering and close-up attention elicited tingles in over half of the 450 respondents (Barratt & Davis, 2015).

Although the phenomenology of ASMR overlaps with other atypical conscious states, there are elements of the ASMR experience that appear to be unique. For instance, unlike other sensory-emotional experiences such as *frisson* (i.e., “chills” elicited by music or an emotional experience), which tend to last up to 10 s, individuals with ASMR may experience the tingling sensations induced by a triggering stimulus for several minutes or more (Del Campo & Kehle, 2016; Fredborg, Clark & Smith, 2017). In addition, the intensity and duration of ASMR experiences are often under the control of the individual, a characteristic that distinguishes ASMR from the automatic sensory responses of synesthesia. Indeed, the ability to suppress ASMR responses as they arise may be an important aspect of this phenomenon that warrants further study. Another noteworthy quality of ASMR is that the tingles tend to induce relaxation and positive affect in the individual. The fact that ASMR is associated with both atypical sensory experiences *and* an emotional response suggests that in order to fully understand ASMR, it will be necessary to examine other conditions or experiences that produce similar emotional and sensory changes.

One conscious state that shares some phenomenological characteristics with ASMR is mindfulness (Barratt & Davis, 2015). In their operationalization of mindfulness, Bishop et al. (2004) define the construct as a two-component process by which one engages in both intentional self-regulation of attention and a nonjudgmental awareness and acceptance of the present moment. Other researchers have suggested that mindfulness involves an openness to sensations, attentional control, emotional regulation, and resilience (Kabat-Zinn, 1990). Both of these complementary descriptions of mindfulness overlap with elements of the ASMR experience. For example, the focused attention method of mindfulness meditation requires individuals to focus on a specific external stimulus or internal thought (Lutz et al., 2008). During ASMR experiences, individuals focus attention on an external stimulus that triggers tingling sensations. Both mindfulness and ASMR can lead to a feeling of relaxation that enhances people’s subjective well-being (Barratt & Davis, 2015; Bishop et al., 2004). Given these similarities, an examination of the relationship between ASMR and mindfulness seems warranted.

The current research investigated the relationship between ASMR and both *state* and *trait* mindfulness. State mindfulness reflects a transient, moment-to-moment conscious experience (Tanay & Bernstein, 2013) whereas trait mindfulness is a more stable and enduring tendency to attend to and experience the world in a mindful manner (Brown & Ryan, 2003). Individuals with ASMR and matched control participants completed two well-established self-report measures of mindfulness—the Toronto Mindfulness Scale (TMS), which is more sensitive to state mindfulness (TMS; Lau et al., 2006), and the Mindful Attention and Awareness Scale (MAAS), which is more sensitive to the attentional component of trait mindfulness (MAAS; Brown & Ryan, 2003). ASMR participants also completed the ASMR Checklist (Fredborg, Clark & Smith, 2017), which examines ASMR triggers and the phenomenology of ASMR experiences. We predicted that individuals with ASMR would report higher levels of mindfulness, as measured by both the TMS and the MAAS. We also predicted that individuals with ASMR who reported higher intensity tingles in response to common triggering stimuli would score higher on both the TMS and MAAS than individuals who reported lower intensity tingles. Together, these data would provide novel insight into the phenomenology of ASMR, while also showing how this atypical conscious state is related to mindful states and traits.

## Methods and Materials

### Participants

The current study is the second part of a two-part study that examined personality traits that co-occur with ASMR (Fredborg, Clark & Smith, 2017). A total of 284 participants who self-reported as experiencing ASMR (*n* = 135 males, *M*_age_ = 28.07, SD = 9.58) and 279 participants who did not indicate ever experiencing ASMR (*n* = 123 males, *M*_age_ = 29.19, SD = 10.55) took part in this study. Given the exploratory nature of this study, an a priori power analysis to determine the ideal sample size for a given effect size would have been based on speculation and was thus not conducted.

Recruitment of participants with ASMR was conducted via an advertisement for the study on a Reddit.com forum dedicated to ASMR (http://www.reddit.com/r/ASMR). As ASMR was largely discovered through discussion on internet forums (Del Campo & Kehle, 2016), we used this recruitment strategy to find as many individuals with ASMR as possible who would be willing to share their experiences online. To gain confidence that individuals recruited to the ASMR group genuinely experienced the phenomenon (rather than watched the videos for relaxation purposes), they were asked to answer specific questions about their ASMR experiences when completing a self-created ASMR checklist (Fredborg, Clark & Smith, 2017; as elaborated below). These questions included a list of 14 popular ASMR-triggering stimuli that participants rated both on the intensity of tingling sensations experienced, as well as how many seconds after stimulus onset that they would typically experience tingles. Only participants who completed the entire ASMR checklist were included in the ASMR group during the analyses.

Age- and sex-matched control participants were recruited by the participant recruitment team at *Qualtrics* Panels (Qualtrics, Inc., Provo, UT, USA) which is an internet-based participant recruitment company. Using this recruitment method allowed us to mitigate sampling bias by increasing the likelihood that both ASMR and control participants were regular internet users. All control participants provided informed consent prior to study completion. To ensure that they did not experience ASMR, control participants viewed two popular ASMR-eliciting videos and were asked whether the videos triggered tingling sensations. If an individual indicated that they had experienced ASMR either during the videos or in the past, he or she was guided to an exit screen and thanked for their time. Further details of this screening method are included in the *Procedure* section.

The current study received ethical approval from the University of Winnipeg Department of Psychology’s Human Research Ethics Board.

### Questionnaires

The survey included several well-established questionnaires: the Big Five Inventory (BFI; John, Donahue & Kentle, 1991), the (MAAS; Brown & Ryan, 2003) and the TMS (Lau et al., 2006). It also included several self-created questionnaires: a self-created Embodied Emotion Scale, a self-created ASMR Checklist (Fredborg, Clark & Smith, 2017), and a set of demographic questions to characterize the sample. The results of the relationship between the Big Five personality traits and ASMR have been published separately (Fredborg, Clark & Smith, 2017). The present report focuses on the TMS and the MAAS, and their relationship to data from the ASMR Checklist.

The TMS consists of 13 self-report items that assess experiences of mindfulness immediately preceding a meditation session (Lau et al., 2006). Individuals respond to each item using a five-point Likert-type scale from 0 (“not at all”) to 4 (“very much”). The 13 items on the TMS load onto two subscales. Six items comprise the Curiosity subscale of the TMS, which is a measure of one’s curiosity about any thoughts, emotions, and sensations that arise while engaging in a mindful state. Examples of items on the Curiosity subscale include, “I was more invested in just watching my experiences as they arose, than in figuring out what they could mean,” and, “I remained curious about the nature of each experience as it arose.” The remaining seven items comprise the Decentering subscale, which measures people’s awareness of their own experiences. Items on the Decentering subscale include, “I experienced myself as separate from my changing thoughts and feelings,” and, “I was aware of my thoughts and feelings without over identifying with them” (Lau et al., 2006). In its validation sample, both the Curiosity and Decentering subscales of the TMS demonstrated good levels of internal consistency as measured by Cronbach’s alpha (α = 0.88 and α = 0.84, respectively). The instructions to this questionnaire were slightly modified to be suitable for individuals with ASMR experiences.

The MAAS consists of 15 self-report items that load onto a single trait mindfulness factor (Brown & Ryan, 2003). Items assess people’s ability to attend to and be aware of the present moment, and are rated using a six-point Likert-type scale with response options ranging from “almost always” to “almost never.” Examples of items, which are all reverse-coded, include, “I could be experiencing some emotion and not be conscious of it until sometime later,” and “I rush through activities without being really attentive to them.” Studies have reported that the MAAS has strong psychometric properties; in its validation sample, the authors reported good internal consistency as measured by Cronbach’s alpha (α = 0.82) as well as high levels of convergent and discriminant validity. Similarly, in their study of self-report measures of mindfulness, Baer et al. (2006) reported a Cronbach’s alpha value of 0.86 for this measure.

The ASMR Checklist (Fredborg, Clark & Smith, 2017) consists of questions regarding the ASMR participants’ experiences including the intensity of respondents’ responses to 16 triggers that often elicit ASMR as well as the speed of onset of these tingling sensations. After initial analyses (as described in Fredborg, Clark & Smith, 2017), two of these 16 items were removed because only a very small proportion of the sample reported familiarity with these items. These stimuli items were “watching others sweep” and “watching others refill fountain pens.” As such, 14 popular ASMR-inducing stimuli were analyzed. Individuals were asked to rate each stimulus on a seven-point Likert scale from 0 to 6 (from “no tingles” to the “most intense ASMR experience.”), or were provided the option to choose “unknown” if they were unsure if a stimulus elicited tingles. Additional questions examined the length of time it would take for an individual to experience the tingling sensation after the onset of each of the 14 stimuli, the frequency with which respondents view videos that will elicit ASMR, whether they find ASMR experiences to be pleasurable, and whether they use ASMR videos to relax.

### Procedure

After completing an initial informed consent procedure on the Qualtrics survey, individuals who self-identified as having ASMR were asked to complete five questionnaires (TMS, MAAS, BFI, ASMR Checklist, and a self-created Embodied Emotion Scale) presented in an order that was randomized for each individual. Individuals could save their progress on the questionnaires and return later (within a 2-week period) to complete the study should they need to take a break. After completing the five questionnaires, participants completed a set of standard demographic questions. Upon study completion, participants were thanked for their contribution to ASMR research, and were given the option to provide comments on the study.

Control participants recruited through Qualtrics Panels similarly read and accepted the terms of an informed consent form before beginning the experiment. To ensure their inclusion in the control group, prospective control participants watched a minimum of 5 min of a popular ASMR video embedded in the survey, followed by two questions about their experiences both while watching the video and in daily life. These questions were, “Did you experience tingles while watching the video?” and, “Have you ever experienced intense pleasurable tingles in the head or neck while listening to or watching something mundane happening (e.g., watching someone paint their nails or draw)?” If answered affirmatively, participants were guided to an exit screen and thanked for their time. Participants who did not report experiencing ASMR continued with the study by completing the same four questionnaires (TMS, MAAS, BFI, and a self-created Embodied Emotion Scale) presented in a randomized order, as well as the same set of demographic questions to characterize the sample. Participants in the control group did not complete the ASMR checklist. Upon study completion, Control participants were similarly thanked for their contribution to ASMR research, and were given the option to provide comments on the study.

## Results

The ASMR and Control groups were first compared on the TMS and the MAAS. These comparisons were followed by an analysis of the TMS and the MAAS results in relation to responses on the ASMR Checklist. All analyses were performed using SPSS Statistics for Windows, Version 19.0 (SPSS, Inc., Armonk, NY, USA).

### Toronto mindfulness scale

Scores for the Curiosity and Decentering subscales of the TMS were calculated per their standard scoring protocol (Lau et al., 2006; see Table 1 for descriptive statistics). Reliability analyses for each subscale were run within the ASMR and control groups, respectively. Although the internal consistency, as measured by Cronbach’s alpha, was excellent for the Curiosity subscale across both groups (ASMR participants: α = 0.89, Control participants: α = 0.93), poor reliability was found for the Decentering subscale for ASMR participants (ASMR participants: α = 0.60, Control participants: α = 0.83). Further item-by-item reliability analyses revealed that no single item was unfavorably skewing the internal consistency of the Decentering subscale.

**Table 1:** Mean Mindful Attention Awareness Scale (MAAS) and Toronto Mindfulness Scale (TMS) scores as function of group and gender (SD in parentheses).

	Females		Males	
	ASMR	Controls	ASMR	Controls
MAAS	3.40 (0.79)	3.03 (1.17)	3.28 (0.70)	3.00 (1.17)
TMS—curiosity	15.0 (5.60)	11.9 (6.75)	13.1 (5.70)	12.5 (5.96)
TMS—decentering	11.3 (4.26)	11.6 (5.97)	12.3 (4.54)	13.2 (5.51)

To further explore the poor reliability results of the Decentering subscale, and to adhere to recommendations to run exploratory factor analyses when using established test measures in new contexts (Flake, Pek & Hehman, 2017), a principle components factor analysis (PCA) with Varimax rotations was run on all 13 items of the TMS separately for ASMR and control participants. For ASMR participants, the PCA revealed three factors with eigenvalues greater than 1.0, which in total explained 57.5% of the variance. In contrast to its construction sample (Lau et al., 2006), all Curiosity subscale items and one Decentering item (i.e., “I am more concerned with being open to my experiences than controlling or changing them”) loaded together onto the first factor. The remaining Decentering subscale items were split into two new factors: the first comprised of four items related to acceptance and awareness of thoughts (i.e., “I am aware of my thoughts and feelings without over-identifying with them,” “I approach each experience by trying to accept it,” “I am more invested in just watching my experiences as they arise, than in figuring out what they could mean,” and “I am receptive to observing unpleasant thoughts and feelings without interfering with them”), while the second comprised of two items related to viewing oneself as separate from thoughts (e.g., “I experience myself as separate from my changing thoughts and feelings” and “I experience my thoughts more as events in my mind than as a necessarily accurate reflections of the way things ‘really’ are”). As such, the poor reliability results for the Decentering subscale may, in part, be explained by its split into two distinct factors for ASMR participants.

Consistent with the construction sample, for control participants, a PCA revealed two factors with eigenvalues greater than 1.0, which in total explained 61.8% of the variance. In contrast, the first factor was comprised of all six Curiosity subscale items, as well as two Decentering subscale items (i.e., “I am aware of my thoughts and feelings without over identifying with them,” and “I approach each experience by trying to accept it, no matter if it was pleasant or unpleasant”). The second factor was comprised of four remaining items from the Decentering subscale.

Due to these heterogeneous results from our factor analyses, only the Curiosity subscale of the TMS is reported here, as all items on the Curiosity subscale loaded onto the primary factor of the TMS for both the ASMR and control participants.

As predicted, the ASMR group generated higher scores on the Curiosity subscale of the TMS than did the Control group, *F*(1, 559) = 12.935, MSE = 36.456, *p* < 0.001, η_p_^2^ = 0.023. There was no main effect of Gender on this subscale, indicating no overall differences between Males and Females on Curiosity, *F*(1, 559) = 1.478, MSE = 36.456, *p* = 0.225, η_p_^2^ = 0.003. However, the main effect of Group was qualified by a significant Group by Gender interaction, *F*(1, 559) = 5.746, MSE = 36.456, *p* = 0.017, η_p_^2^ = 0.010. Simple effects analyses revealed that for ASMR participants, Females (*M* = 14.98, SD = 5.60) generated significantly higher scores than Males (*M* = 13.133, SD = 5.69) on Curiosity, *F*(1,559) = 6.624, *p* = 0.01, η_p_^2^ = 0.012. For Control participants, there was no significant difference between Males and Females on this subscale, *p* = 0.407. Thus, our results indicate that females with ASMR have significantly higher levels of the Curiosity sub-facet of mindfulness than males with the condition.

### Mindful attention awareness scale

The total score on the MAAS was computed according to a standard scoring protocol (Brown & Ryan, 2003). Consistent with previous research (Baer et al., 2006; Brown & Ryan, 2003), reliability analyses revealed excellent internal consistency as measured by Cronbach’s alpha (ASMR participants: α = 0.85, Control participants: α = 0.95). Overall, the MAAS and the TMS Curiosity subscale were modestly positively correlated, *r*(563) = 0.234, *p* < 0.001. However, when analyzed by group (with the total sample of participants per group), the correlation between the MAAS and the TMS Curiosity subscale for the ASMR group was lower for ASMR participants, *r*(284) = 0.132, *p* = 0.026, than Control participants, *r*(279) = 0.265, *p* < 0.001. As predicted, the ASMR group generated higher scores on the MAAS than the Control group, *F*(1, 559) = 15.423, MSE = 0.963, *p* < 0.001, η_p_^2^ = 0.027. There was no main effect of Gender, *F*(1, 559) = 0.835, MSE = 0.963, *p* = 0.361, η_p_^2^ = 0.001, nor a significant interaction between Group and Gender, *F*(1, 559) = 0.421, MSE = 0.963, *p* = 0.516, η_p_^2^ = 0.001.

### ASMR checklist and mindfulness measures

As previously published (Fredborg, Clark & Smith, 2017), reliability analyses revealed good internal consistency for the ASMR Checklist, as indicated by a Cronbach’s alpha of 0.81. A principal-components analysis of the 14 items on the checklist revealed five “intensity” factors underlying the common ASMR-triggering stimuli chosen for the checklist, each of which had an eigenvalue greater than 1.0. These intensity factors (with items in parentheses) were Watching (“Watching others paint,” “Watching others draw,” “Watching others open a package,” and “Watching others cook”), Touching (“Watching someone touch another person’s hair,” “Watching someone touch their own hair,” “Watching others apply makeup and/or nail polish to themselves,” and “Watching others apply makeup and/or nail polish to another person”), Repetitive Sounds (“Tapping sounds” and “Scratching sounds”), Simulations (“Dentist simulation” and “Haircut simulation”), and Mouth Sounds (“Chewing sounds” and “Whispering”), respectively (Fredborg, Clark & Smith, 2017). The average intensity of tingles experienced by all ASMR participants (*N* = 284) in response to the triggering stimuli on the ASMR checklist was calculated and was normally distributed, *M*_Total_ = 2.35, SD = 1.01, *D*(284) = 0.036, NS. Means and standard deviations for the 14 intensity items on this checklist are reported elsewhere (Fredborg, Clark & Smith, 2017, Table 2, p. 5).

**Table 2:** Correlations between the five factors of the ASMR Checklist, average ASMR tingle intensity ratings (*M*_Total_), Toronto Mindfulness Scale Curiosity subscale and Mindful Attention Awareness Scale scores.

	Watching	Touching	Repetitive sounds	Simulations	Mouth sounds	*M*_Total_	MAAS	TMS-C
Watching	1	0.002	−0.18	0.001	−0.009	0.613**	−0.021	0.077
Touching		1	−0.005	0.001	0.009	0.593**	0.115	0.200**
Repetitive sounds			1	0.013	−0.002	0.344**	0.039	0.235*
Simulations				1	0.011	0.289**	0.153*	0.090
Mouth sounds					1	0.254**	−0.056	0.081
*M*_Total_						1	0.112	0.160**
MAAS							1	0.132*^b^
TMS-C								1

**Notes:**

TMS-C refers to the Toronto Mindfulness Scale-Curiosity subscale. MAAS refers to the Mindful Attention Awareness scale.

For all other correlations, *N* = 173 ASMR participants with scores for all checklist items, MAAS items, and TMS-C items.

* Significant at *p* < 0.05.

** Significant at *p* < 0.01.

b *N* = 284 ASMR participants with scores for all MAAS items and TMS-C items.

Scores on the Curiosity subscale of the TMS and the MAAS were correlated with *M*_Total_ and the five intensity factors. The overall relationship between *M*_Total_ and the mindfulness measures was based on the total sample of ASMR participants (*N* = 284). Correlations between the mindfulness measures and the five intensity factors were based only on the 173 ASMR participants who provided numerical intensity ratings to all 14 stimuli (i.e., no stimuli were rated as “unknown”).

Consistent with the prediction that ASMR participants would score higher than controls on the Curiosity subscale, intensity ratings (*M*_Total_) were positively correlated with Curiosity subscale, *r*(284) = 0.160, *p* = 0.007. This effect appears to be primarily due to the “Touching,” *r*(173) = 0.200, *p* = 0.008, and “Repetitive Sounds,” *r*(173) = 0.235, *p* = 0.002, factors. Scores on the Curiosity subscale were not significantly correlated with any other factor, |*r*|s < 0.09, *p*s > 0.24.

*M*_Total_ intensity ratings also correlated positively with the MAAS, *r*(284) = 0.112, *p* = 0.059, although this result was only marginally significant. This effect is primarily due to the “Simulations” factor, *r*(173) = 0.153, *p* = 0.044. MAAS scores were not significantly correlated with any other factor, |*r*|s < 0.115, *p*s > 0.130. See Table 2 for a list of the correlations between mindfulness scores and ASMR Checklist responses.

#### Additional ASMR checklist measures

Analyses were also performed on the ASMR Checklist items unrelated to individuals’ ASMR triggers. One item asked respondents to indicate whether they experienced chills (i.e., frisson) and whether they were able to distinguish between the phenomenological experiences of ASMR and frisson. The results clearly showed that individuals with ASMR can distinguish between ASMR and frisson; of the 248 individuals with ASMR who identified that they experience frisson (87.3% of respondents with ASMR), 90.7% indicated that ASMR and frisson are distinct perceptual experiences. Furthermore, the experience of frisson within this sample is modestly positively correlated with mindful Curiosity as measured by the Curiosity subscale of the TMS, *r*(284) = 0.131, *p* = 0.027.

The ASMR Checklist also asked respondents to indicate the degree to which ASMR tingles are experienced as pleasurable. On average, 76.1% of respondents indicated that ASMR experiences are “quite pleasurable” and 22.9% of respondents indicated that ASMR experiences are “mildly pleasurable.” The remaining response options—“neutral,” “mildly uncomfortable,” and “quite uncomfortable”—each received one response (0.4% of the total respondents). Correlation analyses revealed that as ratings of ASMR pleasure increased, so did ratings of tingle intensity, *r*(284) = 0.215, *p* < 0.001. Further, ratings of ASMR tingle pleasure were positively correlated with scores on the Curiosity subscale of the TMS, *r*(284) = 0.146, *p* = 0.014.

Finally, ASMR respondents were asked to indicate how often, on average, they use ASMR-triggering videos to relax. Of the 284 ASMR participants surveyed, 5.3% of respondents indicated that they “never” use videos to relax, 6.7% indicated that they use them “less than once a month,” 4.6% indicated that they use them “one a month,” 9.2% use them “2–3 times a month,” 14.1% use them “once a week,” 32.7% use them “2–3 times a week,” and 27.5% use them “daily.” Correlation analyses revealed that the more often people use ASMR-triggering videos to relax, the more intense their tingling experiences, though this was only a modest correlation, *r*(284) = 0.124, *p* = 0.037. Further, a modest positive correlation between the frequency of using ASMR-triggering videos to relax and scores on the Curiosity subscale on the TMS was revealed, though this effect was marginally nonsignificant, *r*(284) = 0.114, *p* = 0.055. In contrast, there was no significant correlation between the frequency of using ASMR-triggering videos to relax and scores on the MAAS, *p* = 0.37.

## Discussion

The current study strongly supports the hypothesis that ASMR is related to mindfulness. Individuals with ASMR generated higher scores on the MAAS, a measure of the attentional component of mindfulness. They also produced higher scores on the Curiosity subscale of the TMS, suggesting a greater interest in and openness to their own conscious experiences. This latter result is also consistent with previous research indicating that individuals with ASMR have higher levels of the openness-to-experience personality trait (Fredborg, Clark & Smith, 2017; Janik McErlean & Banissy, 2017).

The results of the Decentering subscale of the TMS were less clear due to differences in reliability between the groups. Although the internal consistency for the control participants was congruent with previous research (Cronbach’s α = 0.83; Lau et al., 2006), the internal consistency for the ASMR participants was much lower (Cronbach’s α = 0.60). This difference suggests that individuals in the ASMR group did not interpret the questions on this subscale of the TMS in the same way. Some participants may have interpreted these items in a more general (i.e., trait-like) fashion while others interpreted them as being related to the ASMR “tingling” experience (i.e., a state response). Moreover, exploratory factor analyses of the TMS for ASMR participants revealed a three-factor structure in which the Decentering subscale was split into two factors. Given these mixed results, it is problematic to compare the control group to the ASMR group on this subscale. However, the fact that individuals with ASMR reported higher levels of curiosity as well as increased mindful attention suggests that ASMR may be a cognitively “active” process rather than a more automatic response to stimuli.

The current study is not the first to find inter-group differences in the interpretation of items in mindfulness scales. Williams & Dalgleish (2014) found that meditators and nonmeditators differ in their interpretation of some items on the Five-Facet Mindfulness Questionnaire (FFMQ; Baer et al., 2006). These group differences were largest for items relating to the “observe” facet of mindfulness, which focuses on the observation of external and internal stimuli and includes such questions as, “When I’m walking, I deliberately notice the sensations of my body moving.” This component of the FFMQ appears to overlap with the Decentering subscale of the TMS. Given that individuals with ASMR are more mindful than controls, the notion that individuals with ASMR may be more mindfully curious, but equally aware of their experiences, as controls is actually consistent with previous research (Aguado et al., 2015; Williams & Dalgleish, 2014). Further research is necessary to determine whether individuals with ASMR are more or less adept than controls at other facets of mindfulness, such as decentering (e.g., the ability for people to view themselves as separate from their thoughts and feelings).

Additional research is also necessary to characterize the sex differences found in our study. Both male and female ASMR participants scored higher than controls on the MAAS, a measure of the attentional component of ASMR. On the Curiosity subscale of the TMS, however, females with ASMR generated higher scores than males with ASMR. Future studies combining self-report measures with psychophysiological and/or neuroimaging methods would help elucidate the reason(s) for these potential sex differences.

The current study also addressed the relationship between ASMR intensity, specific ASMR triggers, and mindfulness. The overall ASMR intensity score, *M*_Total_, was indeed correlated with the Curiosity subscale of the TMS. This result suggests that individuals who are curious about stimuli will experience ASMR more intensely than less-curious individuals. This, again, suggests that ASMR is a cognitively active process. The pattern of ASMR triggers also suggests that perceptual curiosity is a factor in ASMR experiences. The “Touching” and “Repetitive Sounds” triggers significantly correlated with Curiosity. It is possible that these types of triggers require more top-down cognitive interpretation than triggers such as whispering. Listening to repetitive sounds and watching people touch things may require the observer to create a mental structure of the perceived stimuli. Individuals who are proficient at doing so—or who have the motivation or curiosity to do so—would be more likely to experience tingles associated with these stimuli.

The current results raise an interesting question: given that mindfulness is associated with increased ASMR intensity, would mindfulness training lead to enhanced ASMR experiences? The current data suggest that it would, although additional research is necessary to test this hypothesis. If such a study produced positive results, it would present individuals with ASMR with some interesting possibilities. Many individuals with ASMR enjoy “tingles” hedonically or as a form of relaxation, whereas others use ASMR to help reduce the intensity of some of the symptoms associated with anxiety and depression (Barratt & Davis, 2015). If mindfulness training were to enhance the subjective effects of ASMR, it is possible that this training might enhance any potential benefits that ASMR has on subjective well-being. However, these possible benefits of ASMR are, at present, speculative. To date, the positive impact of ASMR on mental and physical health has only been discussed anecdotally and in self-report survey form. Future research investigating the clinical utility of ASMR, alone and in conjunction with other therapies, would help address this intriguing possibility.

## Conclusions

In conclusion, the results from the Curiosity subscale of the TMS, as well as the MAAS, suggest that the experience of ASMR and mindfulness appear to be related constructs. The current data also indicate that females with ASMR have a higher Curiosity score than males with ASMR, suggesting that the cognitive underpinnings of ASMR may differ between the sexes. Future studies are necessary to thoroughly characterize these mechanisms. Additional research is also necessary to determine whether ASMR could be used to enhance well-being in a manner similar to many mindfulness-based treatment programs.

## Supplemental Information

10.7717/peerj.5414/supp-1 Supplemental Information 1 Mindfulness (TMS and MAAS) scores for all participants and ASMR checklist scores for individuals with ASMR.

10.7717/peerj.5414/supp-2 Supplemental Information 2 The questionnaire used to assess the characteristics of respondents’ ASMR.

## Additional Information and Declarations

### Data Availability

The following information was supplied regarding data availability: The raw data are provided in a Supplemental File.
