# Supplementary material for: Mindfulness and autonomous sensory meridian response (ASMR)
Source: PeerJ. 2018 Aug 7;6:e5414. doi: 10.7717/peerj.5414 (PMC6086079; doi:10.7717/peerj.5414)
Supplement: Supplemental Information 2 [file peerj-06-5414-s002.pdf]

ASMR Checklist

Please evaluate whether or not the following common stimuli trigger your ASMR, and if so, how intense the ASMR experience is on average when engaging with those stimuli.

Intensity Scale (higher numbers represent increasing intensity):
0 = No Tingles
1
2
3 = Moderately Intense
4
5
6 = Most Intense ASMR Experience

or

Unknown

Also, after perceiving a triggering stimulus, please estimate approximately how long it would take for this stimulus to cause tingles.

For example, if whispering triggers the most intense tingles for someone, they would label that stimulus a "6". If it takes approximately 20 seconds after the whispering has started to trigger ASMR, this would also be indicated on the survey using the dropdown box.
**** If you select "unknown" or "0" for stimulus intensity, leave the trigger time blank ****

**Stimulus**	**Intensity (please circle the appropriate number according to the scale above OR select "Unknown")**	**How many seconds after its onset do you feel tingles? (please circle one or select "Unknown")**
Whispering	0 1 2 3 4 5 6<br>Unknown	0 – 10 31 – 40<br>11 – 20 41 – 50<br>21 – 30 51 – 60<br>Greater than 60<br>Unknown
Tapping sounds	0 1 2 3 4 5 6<br>Unknown	0 – 10 31 – 40<br>11 – 20 41 – 50<br>21 – 30 51 – 60<br>Greater than 60<br>Unknown
Scratching sounds		0 – 10 31 – 40<br>11 – 20 41 – 50

	0 1 2 3 4 5 6<br>Unknown	21 – 30 51 – 60<br>Greater than 60<br>Unknown
Chewing sounds	0 1 2 3 4 5 6<br>Unknown	0 – 10 31 – 40<br>11 – 20 41 – 50<br>21 – 30 51 – 60<br>Greater than 60<br>Unknown
Haircut simulation	0 1 2 3 4 5 6<br>Unknown	0 – 10 31 – 40<br>11 – 20 41 – 50<br>21 – 30 51 – 60<br>Greater than 60<br>Unknown
Dentist simulation	0 1 2 3 4 5 6<br>Unknown	0 – 10 31 – 40<br>11 – 20 41 – 50<br>21 – 30 51 – 60<br>Greater than 60<br>Unknown
[Optional] Other simulation, please specify if applicable: ____________________ __________	0 1 2 3 4 5 6<br>Unknown	0 – 10 31 – 40<br>11 – 20 41 – 50<br>21 – 30 51 – 60<br>Greater than 60<br>Unknown
Watching others open a package	0 1 2 3 4 5 6<br>Unknown	0 – 10 31 – 40<br>11 – 20 41 – 50<br>21 – 30 51 – 60<br>Greater than 60<br>Unknown
Watching others refill fountain pens	0 1 2 3 4 5 6<br>Unknown	0 – 10 31 – 40<br>11 – 20 41 – 50<br>21 – 30 51 – 60<br>Greater than 60<br>Unknown

Watching others apply makeup and/or nail polish to another person	0 1 2 3 4 5 6<br>Unknown	0 – 10 31 – 40<br>11 – 20 41 – 50<br>21 – 30 51 – 60<br>Greater than 60<br>Unknown
Watching others apply makeup and/or nail polish to themselves	0 1 2 3 4 5 6<br>Unknown	0 – 10 31 – 40<br>11 – 20 41 – 50<br>21 – 30 51 – 60<br>Greater than 60<br>Unknown
Watching someone touch another person’s hair	0 1 2 3 4 5 6<br>Unknown	0 – 10 31 – 40<br>11 – 20 41 – 50<br>21 – 30 51 – 60<br>Greater than 60<br>Unknown
Watching someone touch their own hair	0 1 2 3 4 5 6<br>Unknown	0 – 10 31 – 40<br>11 – 20 41 – 50<br>21 – 30 51 – 60<br>Greater than 60<br>Unknown
Watching others draw	0 1 2 3 4 5 6<br>Unknown	0 – 10 31 – 40<br>11 – 20 41 – 50<br>21 – 30 51 – 60<br>Greater than 60<br>Unknown
Watching others paint	0 1 2 3 4 5 6<br>Unknown	0 – 10 31 – 40<br>11 – 20 41 – 50<br>21 – 30 51 – 60<br>Greater than 60<br>Unknown
Watching others cook	0 1 2 3 4 5 6<br>Unknown	0 – 10 31 – 40<br>11 – 20 41 – 50<br>21 – 30 51 – 60<br>Greater than 60<br>Unknown

Watching others sweep	0 1 2 3 4 5 6<br>Unknown	0 – 10 31 – 40<br>11 – 20 41 – 50<br>21 – 30 51 – 60<br>Greater than 60<br>Unknown
[Optional] Watching any other mundane task, please specify if applicable:<br>____________________________	0 1 2 3 4 5 6<br>Unknown	0 – 10 31 – 40<br>11 – 20 41 – 50<br>21 – 30 51 – 60<br>Greater than 60<br>Unknown
[Optional] Any specific scent, if applicable:<br>______________________________	0 1 2 3 4 5 6<br>Unknown	0 – 10 31 – 40<br>11 – 20 41 – 50<br>21 – 30 51 – 60<br>Greater than 60<br>Unknown
[Optional] Any other notable trigger, please specify if applicable:<br>_______________________	0 1 2 3 4 5 6<br>Unknown	0 – 10 31 – 40<br>11 – 20 41 – 50<br>21 – 30 51 – 60<br>Greater than 60<br>Unknown
[Optional] Any other notable trigger, please specify:<br>_______________________	0 1 2 3 4 5 6<br>Unknown	0 – 10 31 – 40<br>11 – 20 41 – 50<br>21 – 30 51 – 60<br>Greater than 60<br>Unknown
[Optional] Any other notable trigger, please specify:<br>_______________________	0 1 2 3 4 5 6<br>Unknown	0 – 10 31 – 40<br>11 – 20 41 – 50<br>21 – 30 51 – 60<br>Greater than 60<br>Unknown

Are your experiences more intense when the stimulation is directed *toward you* (e.g., someone whispering to you)? Y / N / Unsure

Is your ASMR experience more intense when you observe someone performing an action to/for themselves (e.g., applying their own makeup) rather than when you observe someone acting upon another person (e.g., applying makeup to someone else)? Y / N / Unsure

How often do you use ASMR videos or audio files to help you go to sleep?

a) Never
b) Less than Once a Month
c) Once a Month
d) 2 – 3 Times a Month
e) 2 – 3 Times a Week
f) Daily

How often do you use ASMR videos or audio files to help you relax (but not sleep)?

a) Never
b) Less than Once a Month
c) Once a Month
d) 2 – 3 Times a Month
e) 2 – 3 Times a Week
f) Daily

Have you ever experienced "chills" from beautiful music? Y / N / Cannot Recall

If so, does the ASMR feeling differ getting chills from beautiful music? Y / N

If the ASMR feeling does *NOT* differ from experiencing chills from beautiful music, what genre(s) of music can cause ASMR?

a) Pop
b) Country
c) Hip-Hop
d) Jazz
e) Classical
f) Choral Music
g) Other, please specify: ____________________

On average, how pleasurable is an ASMR experience?

a) Quite Uncomfortable
b) Mildly Uncomfortable
c) Neutral
d) Mildly pleasurable
e) Quite pleasurable

What percent of the time is an ASMR experience *opposite* to how it feels on average? (For example, if ASMR generally feels pleasurable for you, what percent of the time does it feel uncomfortable?)

a) 50 percent of the time.
b) Greater than 25 percent of the time, but less than 50 percent of the time.
c) Greater than 10 percent of the time, but less than 25 percent of the time.
d) Between 1 and 10 percent of the time.
e) ASMR virtually always feels the way I specified in the previous question.

What is the earliest age you can recall having an ASMR experience? (For example, if you remember first having an ASMR experience at the age of four, please choose "4".

In this question, a "first ASMR experience" refers to the first time that you noticed you were experiencing ASMR-related tingles, even if at the time you did not know what they were called.

If you aren't sure, make your best guess.

___ years old

Have you ever had a seizure? Y / N / Unsure

If yes, approximately how many have you had? ______________________________
